# Supplementary material for: Teaching Urology to Undergraduates: A Prospective Survey of What General Practitioners Need to Know
Source: Int J Environ Res Public Health. 2021 Nov 7;18(21):11687. doi: 10.3390/ijerph182111687 (PMC8583650; doi:10.3390/ijerph182111687)
Supplement: Supplementary file 1 [file ijerph-18-11687-s001.zip › Supplementary Figure S1.pdf]

TITLE PAGE

Teaching urology to undergraduates: A prospective survey of what General Practitioners need to know

Borque-Fernando, Á[1,2]; Redondo Redondo, C.[1,2]; Orna Montesinos, C.[3]; Esteban Escaño, L.M.[4]; Denizón Arranz, S. [5]; Tejero Sánchez, A.[2]; García Ruiz, R.[2]; Sánchez Zalabardo, J.M. [2]; Gracia Romero, J. [1,2]; Monreal Hijar, A. [6]; Gil Sanz, M.J. [1,2]; Sanz Saiz, G. [7]; Sanz Pozo, M.[2]; Romero Fernández, F. [1]

[1]Department of Surgery, Gynaecology and Obstetrics. Urology area. School of Medicine, University of Zaragoza (Spain).

[2]IIS Aragon

[3]Department of English and German Philology. School of Education. University of Zaragoza (Spain)

[4]Polytechnic University School la Almunia de Doña Godina, University of Zaragoza (Spain).

[5]Faculty of Health Sciences. University Francisco de Vitoria, Madrid (Spain)

[6]Department of Medicine, Psychiatry and Dermatology. Medicine Area. School of Medicine, University of Zaragoza (Spain)

[7]Department of Statistical Methods. Area of Statistics and Operational Research. Faculty of Science, University of Zaragoza (Spain)

UROLOGICAL TRAINING REQUIREMENTS IN PRIMARY HEALTH CARE

**Experience:**	**Gender :** O Male O Female
**Workplace:** O Urban O Rural	**Healthcare area:** O Primary Health care O Emergency
**Medical speciality** O General Practitioner O Pediatrics O General Practitioner Intern O Pediatric Intern	

In the following questions, circle the number according to your opinion about the importance of the subject. Although some aspects are not related with the speciality of Urology, they are used as a comparative control with other diseases.

**Which level of knowledge do you consider is required by a GP/ pediatrician, to deal with the next subjects ?**	**Level of knowledge**				
	**Very low**	**Low**	**Intermediate**	**High**	**Very high**
**Congenital disorders**	1	2	3	4	5
**PSA levels and screening for prostate cancer**	1	2	3	4	5
**Headache**	1	2	3	4	5
**Renal colic**	1	2	3	4	5
**Testicular cancer: *diagnosis***	1	2	3	4	5
**Testicular cancer: *staging and disease management***	1	2	3	4	5
**Huntington's disease**	1	2	3	4	5
**Erectile dysfunction**	1	2	3	4	5
**Bladder cancer: *diagnosis***	1	2	3	4	5
**Bladder cancer: *staging and disease management***	1	2	3	4	5
**Scrotal pain**	1	2	3	4	5
**Renal cell cancer: *diagnosis***	1	2	3	4	5
**Renal cell cancer: *staging and disease management***	1	2	3	4	5
**Pheochromocytoma**	1	2	3	4	5
**Phimosis**	1	2	3	4	5
**Haematuria**	1	2	3	4	5
**Lower urinary tract symptoms/benign prostatic hyperplasia**	1	2	3	4	5
**Enuresis**	1	2	3	4	5
**Hypertension**	1	2	3	4	5
**Urinary incontinence**	1	2	3	4	5
**Upper respiratory tract**	1	2	3	4	5

[illegible]039 (Coordinador: Á. Borque)

**infections**					
**Urological Infections**	1	2	3	4	5
**Male infertility**	1	2	3	4	5
**Penile cancer: *diagnosis***	1	2	3	4	5
**Penile cancer: staging and disease management**	1	2	3	4	5
**Vesicoureteric reflux**	1	2	3	4	5
**Lung transplantation**	1	2	3	4	5
**Prostate cancer: diagnosis**	1	2	3	4	5
Prostate cancer: staging and disease management	1	2	3	4	5
**Renal transplantation**	1	2	3	4	5
**Urogenital trauma**	1	2	3	4	5
**Vasectomy**	1	2	3	4	5
**Neurogenic bladder dysfunction**	1	2	3	4	5

**Which level of knowledge do you consider is required by a GP/ pediatrician, to deal with the next subjects ?**	**Level of knowledge**				
	**Very low**	**Low**	**Intermediate**	**High**	**Very high**
**Pulmonary auscultation**	1	2	3	4	5
**Cystoscopy**	1	2	3	4	5
**Laparoscopic cholecystectomy**	1	2	3	4	5
**Interpretation of urine test (urine sediment and culture)**	1	2	3	4	5
**Percutaneous nephrostomy placement**	1	2	3	4	5
**Semen analysis**	1	2	3	4	5
**Urodynamic tests**	1	2	3	4	5
**Transurethral catheterisation**	1	2	3	4	5
**ECG interpretation**	1	2	3	4	5
**Abdominal examination**	1	2	3	4	5
**Suprapubic cystostomy catheterisation**	1	2	3	4	5
**Drainage of a cutaneous abscess**	1	2	3	4	5
**Dental examination**	1	2	3	4	5
**Swan-Ganz catheter placement**	1	2	3	4	5
**Genital examination**	1	2	3	4	5
**Lumbar punction**	1	2	3	4	5

**EEG interpretation**	1	2	3	4	5
**Digital rectal examination**	1	2	3	4	5
**Evaluation of imaging techniques (excretory urography, CT, MRI, US)**	1	2	3	4	5

**To sum up…**	**Very low**	**Low**	**Intermediate**	**High**	**Very high**
**Rate the importance of urological diseases in your professional practice**	1	2	3	4	5

¿Are you an associate Professor of Medicine at the University: ○ YES ○ NO

Thank you for your contribution

PIIDUZ_13_039 (Coordinador: A. Borque)
