# Supplementary material for: Teaching Urology to Undergraduates: A Prospective Survey of What General Practitioners Need to Know
Source: Int J Environ Res Public Health. 2021 Nov 7;18(21):11687. doi: 10.3390/ijerph182111687 (PMC8583650; doi:10.3390/ijerph182111687)
Supplement: Supplementary file 1 [file ijerph-18-11687-s001.zip › Supplementary Table S1.pdf]

TITLE PAGE

Teaching urology to undergraduates: A prospective survey of what General Practitioners need to know

Borque-Fernando, Á[1,2]; Redondo Redondo, C.[1,2]; Orna Montesinos, C.[3]; Esteban Escaño, L.M.[4]; Denizón Arranz, S. [5]; Tejero Sánchez, A.[2]; García Ruiz, R.[2]; Sánchez Zalabardo, J.M. [2]; Gracia Romero, J. [1,2]; Monreal Hijar, A. [6]; Gil Sanz, M.J. [1,2]; Sanz Saiz, G. [7]; Sanz Pozo, M.[2]; Romero Fernández, F. [1]

[1]Department of Surgery, Gynaecology and Obstetrics. Urology area. School of Medicine, University of Zaragoza (Spain).

[2]IIS Aragon

[3]Department of English and German Philology. School of Education. University of Zaragoza (Spain)

[4]Polytechnic University School la Almunia de Doña Godina, University of Zaragoza (Spain).

[5]Faculty of Health Sciences. University Francisco de Vitoria, Madrid (Spain)

[6]Department of Medicine, Psychiatry and Dermatology. Medicine Area. School of Medicine, University of Zaragoza (Spain)

[7]Department of Statistical Methods. Area of Statistics and Operational Research. Faculty of Science, University of Zaragoza (Spain)

Supplementary file 1. Reliability analysis

Item	Alpha	Std.Alpha	r(item,total)
V6	0,935	0,931	-0,213
V7	0,93	0,928	0,381
V8	0,926	0,925	0,755
V9	0,927	0,925	0,766
V10	0,928	0,926	0,547
V11	0,926	0,924	0,796
V12	0,927	0,925	0,655
V13	0,927	0,925	0,7
V14	0,927	0,925	0,689
V15	0,928	0,926	0,568
V16	0,927	0,925	0,643
V17	0,928	0,926	0,653
V18	0,93	0,928	0,19
V19	0,929	0,927	0,438
V20	0,929	0,926	0,5
V21	0,926	0,924	0,831
V22	0,93	0,928	0,288
V23	0,931	0,929	0,097
V24	0,928	0,926	0,549
V25	0,932	0,93	-0,014
V26	0,93	0,927	0,389
V27	0,93	0,927	0,445
V28	0,93	0,928	0,293
V29	0,929	0,927	0,406
V30	0,934	0,931	-0,161
V31	0,93	0,928	0,216
V32	0,926	0,924	0,812
V33	0,93	0,928	0,301
V34	0,929	0,927	0,442
V35	0,928	0,925	0,668
V36	0,929	0,926	0,473
V37	0,927	0,925	0,736
V38	0,927	0,925	0,692
V39	0,932	0,933	-0,363
V40	0,929	0,927	0,472
V41	0,927	0,925	0,661
V42	0,928	0,925	0,663
V43	0,928	0,925	0,594
V44	0,929	0,926	0,499
V45	0,934	0,932	-0,245
V46	0,93	0,926	0,449
V47	0,929	0,926	0,44
V48	0,928	0,926	0,612
V49	0,929	0,927	0,456
V50	0,93	0,929	0,2
V51	0,929	0,926	0,482
V52	0,928	0,926	0,591
V53	0,929	0,927	0,432
V54	0,928	0,926	0,586
V55	0,934	0,931	-0,183

V56	0,93	0,929	0,221
V57	0,93	0,928	0,27
V58	0,93	0,928	0,339
